# Midseptal and Anteroseptal Accessory Pathway Ablation in Children

**DOI:** 10.3390/jcm13226885

**Published:** 2024-11-15

**Authors:** László Környei, Matevž Jan, Mohammad Ebrahim, Vjekoslav Radeljić, Mirta Rode, Diana Delić-Brkljačić, Ivana Kralik, Flóra Kocsis, Nikola Krmek

**Affiliations:** 1 Gottsegen National Cardiovascular Center, Hungarian Paediatric Heart Center, 1096 Budapest, Hungary; drkornyei@gmail.com (L.K.); florakocsis.dr@gmail.com (F.K.); 2 University Medical Centre Ljubljana, 1000 Ljubljana, Slovenia; matevz.jan@kclj.si; 3 Chest Diseases Hospital, Kuwait University, Kuwait City 46300, Kuwait; mohdi84@gmail.com; 4 University Hospital Center Sestre Milosrdnice, School of Medicine, University of Zagreb, 10000 Zagreb, Croatia; vjekoslav.radeljic@gmail.com (V.R.); mirta.rode@kbcsm.hr (M.R.); diana.delic.brkljacic@kbcsm.hr (D.D.-B.); 5 Dubrava University Hospital, 10000 Zagreb, Croatia; ikralik@kbd.hr

**Keywords:** accessory pathway, electrophysiology, radiation dose, cryoablation, heart block

## Abstract

**Objectives:** The goal of this study is to document outcomes of ablation for high-risk accessory pathways in paediatrics using 3D mapping systems with minimal to zero fluoroscopy. **Methods:** It is a cross-sectional, multicentre study, conducted between 2013 and 2023, and involving four different centres in Hungary, Croatia, Kuwait, and Slovenia. **Results:** A total of 128 procedures were performed on 111 patients. The cohort included 57.8% anteroseptal (AS) pathways and 42.2% midseptal (MS) pathways. The mean follow-up time was 2.0 ± 2.1 years. Cryoablation was used in 72.7% of the cases, and radiofrequency ablation was used in 27.3%. The EnSite Precision™ Cardiac Mapping System was the predominant system used. The overall acute success rate was 89.1%, with recurrence rates at 17.2% with similar results regardless of the type of energy used. The success rate was not significantly different between AS and MS substrates. The age and weight of the patient had no bearing on the outcomes (median age and weight were 13 years and 52 kg, respectively). The complications rate was at 4.69% and included transient AV block (three patients), hematoma (one patient), right bundle branch block (one patient), and possible permanent complete AV block (one patient). Fluoroscopy was utilized in 18 cases, with a fluoroscopy time mean of 3 min and 45 s. **Conclusions:** MS and AS AP in paediatric patients can be treated effectively with either RF or cryoablation and with a low dose of radiation using 3D mapping systems, with excellent acute success rates and low complication rates.

## 1. Introduction

Transcatheter ablation of accessory pathways (AP) is the most common electrophysiology (EP) procedure performed in the paediatric population with an overall success rate of over 90% [1,2,3]. AP connects the atrium and the ventricle parallel to the conduction tissue of the AV node and the bundle of His, and can be located in the myocardial walls of the right or left ventricle or in the interventricular septum. At the beginning of an EP study, the characteristics of the AP should be determined, followed by mapping preferably using 3D mapping systems to reduce the use of fluoroscopy and the stochastic risks of ionizing radiation. Ablation is possible on all sites, but is most challenging when performed on pathways located near the normal conduction system due to the risk of inadvertent AV block [4,5,6].

**Figure 1:**
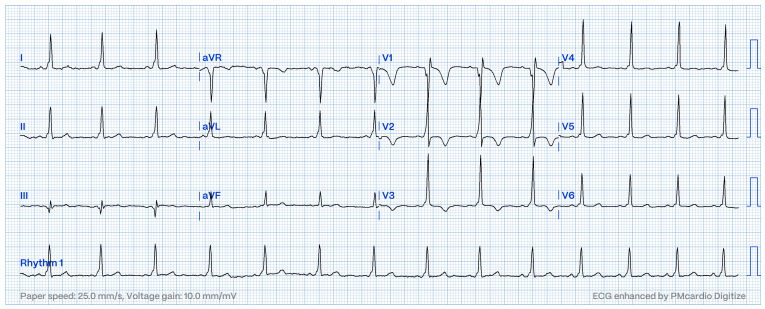
ECG in a patient with a midseptal accessory pathway.

**Figure 2:**
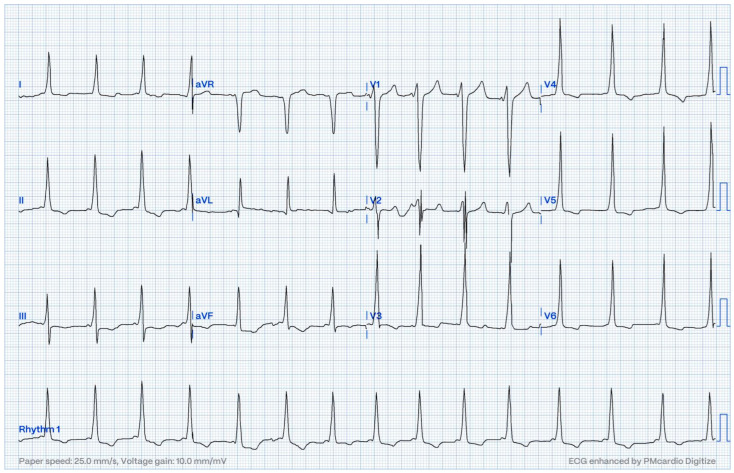
ECG in a patient with an anteroseptal accessory pathway.

Midseptal (MS) and anteroseptal (AS) pathways are uncommon pathways that should be approached with extreme caution [4,7] (Figure 1 and Figure 2). MS APs are located in the only true muscular septal area between the mitral and tricuspid valve fulcrums, which corresponds to the location of the triangle of Koch and the AV node. AS pathways are located above and anterior to the His bundle recording site [8].

Both radiofrequency (RF) and cryoablation can be used for ablation at sites near the conduction system. Cryoablation, with the option of reversible cryomapping and its good adherence to surrounding tissue, reduces the risk of AV block, and is therefore often used for the ablation of septal APs [9]. RF ablation has a high success rate but presents a greater risk of damaging the normal conduction system [10]. General complications of both types of catheter ablation in children include AV block, heart perforation, pericardial effusion, thrombi, emboli, pseudoaneurysms, arterio-venous fistula formation, minor bleeding, and direct injury of adjacent structures (i.e., the oesophagus, the coronary arteries, the phrenic nerve) [9].

The risks of stochastic effects of ionizing radiation are more significant in children than in adults [11]. Locating the arrhythmia substrate can be achieved through fluoroscopy guidance or by using 3D mapping. The zero fluoroscopy approach is a safe and beneficial option for both RF and cryoablation in the paediatric population [12,13]. Dose area product (DAP) is used to quantify the irradiation of patients and it is usually measured by the DAP-meter as an integrated part of the X-ray unit [14].

The goal of this study was to analyse electrophysiological studies with ablation of MS and AS AP with regards to several parameters including success rate, radiation dose, length of the procedure, and modality of energy used. There are limited studies focusing on outcomes of high-risk septal pathways ablation in paediatrics, especially without the use of fluoroscopy. We aim to point out the differences in practice that the 3D mapping systems have brought in this era with as low as reasonably achievable use of X-rays. Few studies have described the outcomes in paediatric patients with such APs [10,13,15,16,17,18,19,20,21].

## 2. Materials and Methods

This is a multicentre cross-sectional study from 4 hospitals: Gottsegen National Cardiovascular Center Budapest, Hungary, Sestre milosrdnice University Hospital Center, Zagreb, Croatia, Chest Diseases Hospital, Kuwait University, Kuwait City, Kuwait, and Ljubljana University Medical Centre, Ljubljana, Slovenia.

Our research included all paediatric patients with MS or AS AP who underwent EP study and ablation between January 2013 and May 2023.

The data were collected from patient’s records and included gender, age at the time of intervention, weight, arrhythmia substrate, indication for EP study, mapping system used, type of ablation, procedure time, fluoroscopy time, DAP, outcome, and complications.

Indications for the EP study were symptomatic (Wolff–Parkinson–White syndrome—WPW) or asymptomatic preexcitation caught on the baseline surface ECG, supraventricular tachycardia (SVT), and palpitations clinically attributable to SVT.

If a single patient underwent the procedure multiple times due to recurrence of AS or MS AP, each procedure was analysed separately.

Both local and general anaesthesia were used. However, the ratio between both types of anaesthesia was not analysed. Any antiarrhythmic medications were discontinued before admission. Catheters were placed in the His or the high right atrium area, the coronary sinus (CS) and the right ventricular apex. In most of the procedures, the His area and the high right atrium were mapped with the same catheter according to pathology. The data on the catheter insertion site and size of the cryocatheter were not available for all patients, therefore this information was not included in this study. Mostly, the CS catheter was placed using the jugular approach; the remaining catheters were placed through femoral veins. The EnSite Precision^®^™ Cardiac Mapping System V 2.6.1 (Abbott) or CARTO^®^ 3 system V 8.1 (Biosense Webster, Johnson & Johnson) were utilized for visualization and 3D mapping, while fluoroscopy was utilized occasionally in certain circumstances, such as in challenging vein punctures, in checking the position with difficulties in catheter placements, or for coronary artery and coronary sinus angiography. Ultrasound guidance for vein or artery puncture and intracardiac echocardiography were used according to the operator’s preference.

**Figure 3:**
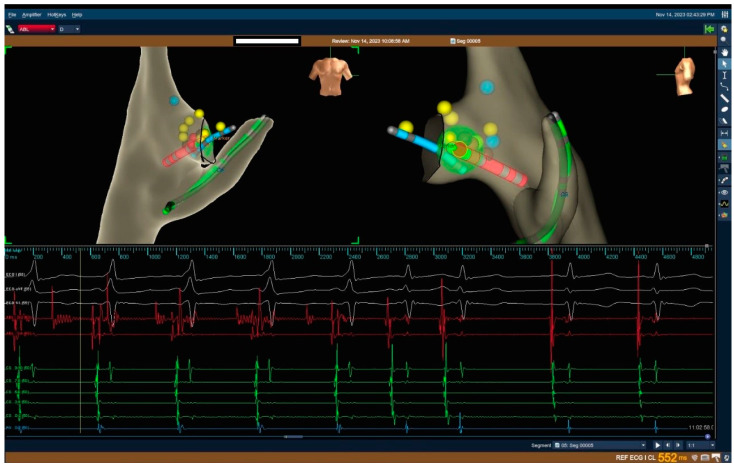
A 3D map, surface, and intracardiac electrograms from The EnSite Precision^®^™ Cardiac Mapping System of a 10-year-old child during cryoablation of a para-Hisian AP (dark dot—catheter bump, red dots—cryolesions, yellow dots—His signals).

Atrial and ventricular programmed stimulation were carried out at baseline according to standard protocols. If needed, it was repeated after the administration of isoproterenol, during the washout phase of isoproterenol, and with atropine. Using the aforementioned 3D mapping systems, the ablation site was determined and ablation was performed using RF or cryoablation depending on the operator’s preference (Figure 3). If cryoablation was the chosen modality, cryomapping was performed on each ablation site before ablation. The complications that occurred during the procedures were noted and analysed.

Acute success was determined either with loss of preexcitation, lack of retrograde pathway conduction, and/or inability to induce SVT (if it was inducible before ablation). In cases of doubt, a negative adenosine test was used as a confirmation that an AP was no longer present. The procedure was deemed acutely successful if these conditions persisted during the first 30 min after ablation. Recurrences were defined as a return of preexcitation or occurrence of SVT and were identified during follow-up.

Several statistical analysis tools were used in this study. Initially, the data underwent analysis utilizing descriptive statistical methods. Subsequently, intergroup differences were examined using an independent sample *t*-test. The χ2 test was then applied to assess the association between categorical variables, while Pearson’s r correlation coefficient was utilized to evaluate the correlation between continuous variables.

## 3. Results

A total of 128 procedures were performed on 111 patients from the four centres. Primary procedures of 109 patients were included, 17 of them underwent a redo procedure, and 2 patients underwent a redo procedure after a failed primary procedure in other centres. A total of 76 procedures from Hungary, 21 from Croatia, 21 from Slovenia, and 10 from Kuwait are included in this study. Based on the final diagnosis, 74 procedures were classified as an AS AP ablation (57.8%), and 54 procedures as a MS AP ablation (42.2%) (Table 1). The mean follow-up time was 2.0 ± 2.1 years.

Two procedures were performed using the CARTO™ system, while the others were performed using the EnSite Precision™ Cardiac Mapping System. Cryoablation was used in 72.7% of the cases, and RF ablation was used in 27.3%. The frequency of utilization of each energy modality was not significantly different between the AS and MS substrates (*p* = 1).

More than half of the patients (58.6%) had symptoms before the procedure. Specifically, symptoms were present in 63% of patients with MS AP, and 55% of patients with AS AP. The most common indication for an EP study was symptomatic WPW syndrome (51.6%), followed by asymptomatic preexcitation (21.1%), palpitations (14.8%), and a documented SVT prior to the study (12.5%).

The overall acute ablation success rate was 89.1%. The success rate was not significantly different between AS and MS substrates (*p* = 0.73), nor between the primary and redo procedures (*p* = 0.74). Acute success was similar between procedures performed with RF and cryoablation energy (86% and 90%, respectively), without a statistical difference (*p* = 0.67).

The outcomes were not related to the age of the patient (*p* = 0.16), nor to the patient’s weight (*p* = 0.09).

**Figure 4:**
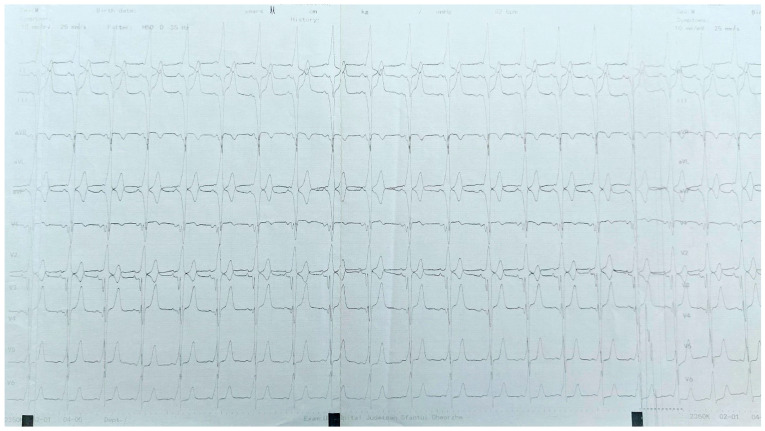
ECG before the EP study and ablation with full preexcitation.

**Figure 5:**
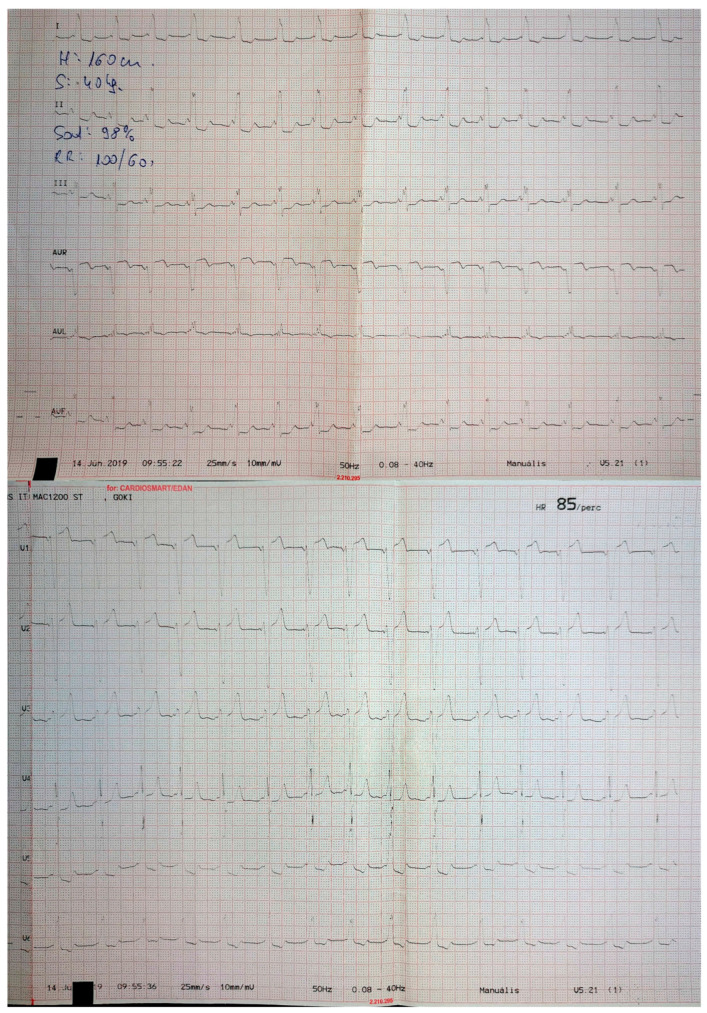
ECG with full preexcitation after cryoablation which resulted in a complete AV block.

Complications were reported in 4.69% of the procedures, and they included transient AV block (three patients—5, 9, and 15 years of age), hematoma of the site of vascular access (13 years of age), RBBB (15 years of age), and a possible complete AV block (11 years of age). The latter occurred in a patient with full preexcitation (Figure 4). Mapping showed a posteroseptal pathway, but during irrigated RF ablation of this pathway preexcitation changed, and an AS pathway became manifest. Cryomapping was able to block the AS pathway separately but after 2 × 4 min of cryoablation, the pathway conduction returned with full preexcitation. A total of 11 cryomapping applications on this successful location with −30 °C for 30–40 s were ineffective. Presuming myocardial edema, an additional nine cryoablation applications with −70 °C for 20 s were delivered for mapping purposes when suddenly complete AV block developed with junctional escape rhythm. After a few hours, pathway conduction partially recurred but appeared unreliable based on the intermittent transient AV block. Five days later, a VVI pacemaker was implanted. On further follow-up, the pacemaker interrogation showed no pacing activity, and the previous frequent tachycardia resolved. The patient became asymptomatic without any antiarrhythmic medication but with full preexcitation on ECG (Figure 5), so a permanent AV block could not be excluded.

Recurrence was detected during follow-up in 17.2% of patients. There was no difference between the frequency of recurrences after the primary or the redo procedures (*p* = 1), nor was there a correlation between recurrences of AP and the energy modality used (*p* = 0.79).

The mean procedure time was 2 h and 21 min (range 1 h to 6 h). Procedure time was not correlated to the weight of the patient (*p* = 0.92). The *t*-test for independent samples determined there was no statistically significant difference in the duration of the procedure between the first and redo procedures (*p* = 0.61). On the other hand, the *t*-test showed that the group with RF ablation had on average a significantly shorter duration of the procedure than with cryoablation (*p* = 0.00) (Table 2).

Fluoroscopy was utilized in 18 procedures (14%), with a fluoroscopy time mean of 3 min and 45 s (SD 4 min 41 s) and a median time of 1 min and 19 s (IQR 16 s—5 min 16 s). The average DAP was 22.01 cGycm2 (SD 41.57), and the median DAP was 7.18 cGycm2 (IQR 1.05–21.28) (Table 3). There is no statistically significant difference in the fluoroscopy time between the first and redo procedures (*p* = 0.29) and between RF and cryoablation groups (*p* = 0.73).

## 4. Discussion

This multicentre cross-sectional study is the first report for both MS and AS AP ablations in the paediatric population performed with the use of 3D mapping systems.

The overall procedural acute success rate of 89.1% and recurrence rate of 17.2%, was similar to the results in the study of Kovach et al. [15] which included cases done exclusively with fluoroscopy. They described acute success in 87% and recurrence in 18% of patients. Similar to our report, there was no difference in success rates between RF and cryoablation. Furthermore, we found similar success rates in repeat and primary procedures.

In a large retrospective national database analysis, Walsh et al. [2] have shown a success rate of 92% with a recurrence rate of 12% of ablation procedures for AP in all possible locations. The acute success rate in our study was lower and the recurrence rate was higher; however, considering that MS and AS pathways have the most challenging location, we believe the differences are minor and acceptable. Importantly, our study did not suggest an increased risk of repeat procedure after cryoablation, which was described by Walsh et al. [2].

In this report, the age and the weight of the patients did not affect the success rates, and the duration of the procedures was not different in smaller individuals, bearing in mind that the smallest child in our study weighed 12.5 kg. As such, we argue against delaying the procedure for older age.

The MAP-IT registry analysis by Dubin et al. [22] showed that RF energy was overall more frequently used for atrioventricular nodal reentry tachycardia (AVNRT) and atrioventricular reentry tachycardia (AVRT) ablation. They have also shown that 26% of all procedures were performed with cryoablation, but when analysing septal pathways separately, it was most commonly used. This is consistent with our analysis, where cryoablation was utilized in 72.7% of procedures. In a study by Dionne et al. [23], cryoablation of AP resulted in a higher recurrence rate compared to RF ablation. However, Jiang et al. [16] reported a low recurrence rate of 13% for both AS and MS AP when using cryoablation only. Similarly, Swissa et al. [17] described a recurrence rate of para-Hisian AP cryoablation in children of 14.9%. Our experience with both types of ablation shows comparable recurrence rates after RF and cryoablation.

Since the introduction of 3D mapping, the radiation dose (i.e., DAP) in the EP studies has decreased significantly [24,25]. A study by Karadeniz et al. [13], where zero fluoroscopy was used in 90% of procedures, included 43 EP studies with septal AP, including posteroseptal AP. It showed a procedural success rate of 93% with a 12.5% recurrence rate. Similar to this report, 110 out of 128 procedures (86%) included in our study were performed without fluoroscopy. Additionally, there was no significant difference in the use of fluoroscopy between the primary and the repeat procedures.

Compared to two meta-analyses regarding AVNRT ablation [26,27], which demonstrated less fluoroscopy use in cases done with cryoablation, our study did not show longer fluoroscopy times for the RF group. In a retrospective case-controlled study [28] the mean DAP value for AVNRT ablation was 51.4 cGycm^2^, which is much higher compared to our result (7.18 cGy cm^2^).

The MAP-IT registry described a mean fluoroscopy time of 7.0 ± 9.2 min for all EP studies of AVRT and AVNRT in patients < 21 years of age with and without congenital heart disease performed with 3D mapping, and a mean of 6.9 ± 9.8 min only for patients without congenital heart disease [22]. Additionally, Swissa et al. [19] analysed cryoablations of para-Hisian APs in children using the LocaLisa 3D mapping system (Medtronic, Minneapolis, MN, USA), and reported a mean fluoroscopy time of 28.5 ± 23.3 min. Our analysis showed a significantly shorter fluoroscopy time compared to the described data (1.3 min).

The approximate risk of second- and/or third-degree AV block for AS and MS pathways with RF energy ranges from 1% to 3% [9]. The risk of AV block may be higher in younger patients since their Koch’s triangle is smaller than in adults [29]. On the other hand, cryoablation is considered a safe and effective option for the ablation of septal AP in the paediatric population [13,16,17,18,30]. Cryomapping and the stability of the catheter during freezing reduce the risk of permanent AV block [9]. Thus far, there is only one description of a permanent complete AV block following cryoablation of a MS pathway [31]. Permanent first-degree AV block after cryoablation has also been described, but the majority of AV blocks after cryoablation recover during follow-up [16,18]. In our study, we describe another case in a child with WPW syndrome and full preexcitation in which repeated cryoablation at a point that appeared safe may have led to a permanent complete AV block. Even though permanent heart block is possible when using cryoablation, the need for pacemaker insertion is far more common when using RF ablation to treat AS and MS AP [10,32,33].

## 5. Conclusions

This study presents an insight into the ablation of MS and AS AP in children, which can be treated effectively with either RF or cryoablation and with a low dose of radiation using 3D mapping systems. Despite the proven safety of cryoablation, we emphasize that even with this type of ablation, complications such as AV block can occur. According to our data, the success rate is the same regardless of the patient’s age and weight, hence in most cases an ablation procedure may be considered at diagnosis.

## 6. Limitations

There are some limitations related to our study.

Firstly, our clinical practice prioritizes minimizing the use of fluoroscopy to the lowest reasonable extent. Consequently, this study includes only a limited number of cases where fluoroscopy was utilized, diminishing the significance of our conclusions when comparing outcomes with fluoroless procedures. Secondly, this was not a randomized study comparing different energy sources, which introduces a certain bias and reduces the value of conclusions derived from comparison of radiofrequency and cryoablation procedures. Thirdly, the use of intracardiac echocardiography was not reported in our study. However, its use might have influenced outcomes regarding procedural parameters, use of fluoroscopy, acute success rate, and recurrence rate. Fourthly, as already mentioned, sizes of cryocatheter used were not reported, therefore the impact of catheter tip size on outcome could not have been estimated. Lastly, the sites of catheter insertion (i.e., jugular versus femoral) might influence catheter stability and ablation lesion formation; however, this information was not reported in our study, which to some extent diminishes the value of our conclusions.

## Figures and Tables

**Table 1:** Procedures and characteristics of patients.

Procedures	N = 128
Age median (range), years	13.53 (2.21–18.27)
Male/Female (%)	76 (59.4)/52 (40.6)
Height median (range), cm (n = 118)	162.5 (91–188)
Weight median (range), kg (n = 127)	51.8 (12.5–124.8)
Symptomatic before procedure	75 (58.6%)
MS symptomatic (n = 54)	34 (63.0%)
AS symptomatic (n = 74)	41 (55.4%)
Indication for EP study:	
Asymptomatic preexcitation	27 (21.1)
Palpitations	19 (14.8)
Documented SVT	16 (12.5)
WPW syndrome	66 (51.6)
Procedures by chronology:	
First procedure	109 (85.2)
Redo procedure	19 (14.8) *
Arrhythmia substrate:	
AS AP	74 (57.8)
MS AP	54 (42.2)
Type of ablation:	
Cryoablation	93 (72.7)
RF ablation	35 (27.3)
Recurrence rate:	
After the primary procedure (n = 109)	19 (17.4)
After the redo procedure (n = 19)	3 (15.8)
Procedure time median (range) (n = 118)	2 h 5 min 0 s (1–6 h)

* Two patients with the initial procedure at another centre; EP, electrophysiology; SVT, supraventricular tachycardia; WPW, Wolff–Parkinson–White; RF, radiofrequency.

**Table 2:** Procedure time for the first and redo procedure and for the two types of ablation.

	Time		
Procedure Type (n)	Mean	SD	*p*-Value
First (101)	2 H 22 M	1 H 2 M	0.61
Redo (17)	2 H 14 M	1 H 0 M	
Type of Ablation (n)			
RF ablation (30)	1 H 53 M	45 M	0.00
Cryoablation (88)	2 H 30 M	1 H 3 M	

RF, radiofrequency; SD, standard deviation, H, hour; M, minute.

**Table 3:** Radiation parameters (n) for procedures with both 3D mapping and fluoroscopy.

	25. Centile	Median	75. Centile	Mean	SD
Fluoroscopy time (n = 13) (H M S)	0 H 0 M 16 S	0 H 1 M 19 S	0 H 5 M 16 S	0 H 3 M 46 S	0 H 4 M 41 S
DAP (n = 18) (cGycm^2^)	1.05	7.18	21.28	22.01	41.57

DAP, dose area product; SD, standard deviation; H, hour; M, minute; S, second.

## Data Availability

The data presented in this study are available on reasonable request from the corresponding author, after ethical committee approval, due to ethical committee license restrictions.
